# Rietveld refinement of whitlockite-related K_0.8_Ca_9.8_Fe_0.2_(PO_4_)_7_

**DOI:** 10.1107/S1600536810014327

**Published:** 2010-04-24

**Authors:** Igor V. Zatovsky, Ivan V. Ogorodnyk, Nataliya Yu. Strutynska, Nikolay S. Slobodyanik, Nataliya O. Sharkina

**Affiliations:** a Department of Inorganic Chemistry, Taras Shevchenko National University, 64 Volodymyrska str., 01601 Kyiv, Ukraine

## Abstract

The title compound, K_0.8_Ca_9.8_Fe_0.2_(PO_4_)_7_ (potassium deca­calcium iron hepta­phosphate), belongs to the whitlockite family. The structure is built up from several types of metal–oxygen polyhedra: two [CaO_8_], one [CaO_7_] and one [(Ca/Fe)O_6_] polyhedron with a mixed Ca/Fe occupancy in a 0.8:0.2 ratio, as well as three tetra­hedral [PO_4_] units. Of the 18 sites in the asymmetric unit, the site with the mixed Ca/Fe occupation, the K site, one P and one O site are on special positions 6*a* with 3 symmetry, whereas all other sites are on general positions 18*b*. The linkage of metal–oxygen polyhedra and [PO_4_] tetra­hedra *via* edges and corners results in formation of a three-dimensional framework with composition [Ca_9.8_Fe_0.2_(PO_4_)_7_]^0.8−^. The remaining K atoms (site-occupation factor = 0.8) are located in large closed cavities and are nine-coordinated by oxygen.

## Related literature

For the structure of the mineral whitlockite with idealized composition Ca_3_(PO_4_)_2_ (β-polymorph), see: Calvo & Gopal (1975 ▶); Yashima *et al.* (2003 ▶). For KCa_10_(PO_4_)_7_, see: Sandström & Boström (2006 ▶). For powder diffraction investigations and Rietveld refinements of other phosphate-based whitlockites, see: Morozov *et al.* (2000 ▶) for *M* ^I^Ca_10_(PO_4_)_7_ (*M* ^I^ = Li, Na, K); Lazoryak *et al.* (1996 ▶) for Ca_9_Fe(PO_4_)_7_; Morozov *et al.* (2002 ▶) for Ca_9_In(PO_4_)_7_; Strunenkova *et al.* (1997 ▶) for Na_1.5_Ca_9_Fe_0.5_(PO_4_)_7_. For the profile function used in the Rietveld refinement, see: Thompson *et al.* (1987 ▶).

## Experimental

### 

#### Crystal data

K_0.8_Ca_9.8_Fe_0.2_(PO_4_)_7_
*M* *_r_* = 1100.02
Trigonal, 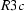

*a* = 10.44282 (1) Å
*c* = 37.29443 (3) Å
*V* = 3522.17 (1) Å^3^
*Z* = 6
Cu *K*α radiation, λ = 1.540598 Å
*T* = 293 K
Flat sheet, 25 × 25 mm

#### Data collection

Shimadzu LabX XRD-6000 diffractometer
Specimen mounting: glass container
Data collection mode: reflection
Scan method: step
2θ_min_ = 8.92°, 2θ_max_ = 99.92°,  increment in 2θ = 0.02°

#### Refinement

*R*_p_ = 8.711
*R*_wp_ = 11.243
*R*_exp_ = 4.919
*R*_Bragg_ = 3.849
*R*(*F*) = 2.48
4551 data points with 839 reflections
131 parameters
4 restraints

### 

Data collection: *PCXRD* (Shimadzu, 2006 ▶); cell refinement: *DICVOL 2004* (Boultif & Louër, 2004 ▶); data reduction: *FULLPROF* (Rodriguez-Carvajal, 2006 ▶); program(s) used to solve structure: *FULLPROF*; program(s) used to refine structure: *FULLPROF*; molecular graphics: *DIAMOND* (Brandenburg, 1999 ▶); software used to prepare material for publication: *PLATON* (Spek, 2009 ▶) and *enCIFer* (Allen *et al.*, 2004 ▶).

## Supplementary Material

Crystal structure: contains datablocks global, I. DOI: 10.1107/S1600536810014327/wm2324sup1.cif

Rietveld powder data: contains datablocks I. DOI: 10.1107/S1600536810014327/wm2324Isup2.rtv

Additional supplementary materials:  crystallographic information; 3D view; checkCIF report

## Figures and Tables

**Table 1:** Selected bond lengths (Å)

Ca1—O11^i^	2.519 (10)
Ca1—O21^ii^	2.702 (13)
Ca1—O22	2.51 (3)
Ca1—O23^ii^	2.40 (2)
Ca1—O32	2.579 (17)
Ca1—O32^iii^	2.57 (2)
Ca1—O33^iii^	2.59 (3)
Ca1—O34	2.48 (3)
Ca2—O12^ii^	2.474 (16)
Ca2—O23^iv^	2.63 (3)
Ca2—O24^iv^	2.444 (19)
Ca2—O24^v^	2.48 (3)
Ca2—O32^v^	2.41 (2)
Ca2—O33^iii^	2.21 (3)
Ca2—O34	2.36 (3)
Ca3—O12	2.295 (15)
Ca3—O21	2.48 (2)
Ca3—O22^vi^	2.49 (3)
Ca3—O23^iv^	2.30 (3)
Ca3—O31	2.38 (3)
Ca3—O31^vii^	2.47 (4)
Ca3—O33^vii^	2.78 (3)
Ca3—O34	2.60 (3)
Ca4—O24	2.30 (3)
Ca4—O31	2.23 (4)
Fe4—O24	2.30 (3)
Fe4—O31	2.23 (4)
K1—O12	2.90 (3)
K1—O21	2.508 (19)
K1—O22	3.25 (3)
P1—O11	1.51 (4)
P1—O12	1.62 (2)
P2—O21	1.49 (2)
P2—O22	1.56 (2)
P2—O23	1.53 (2)
P2—O24	1.486 (17)
P3—O31	1.62 (3)
P3—O32	1.53 (3)
P3—O33	1.57 (3)
P3—O34	1.63 (2)

Symmetry codes: (i) 
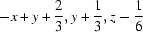
; (ii) 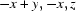; (iii) 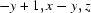; (iv) 
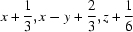
; (v) 
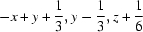
; (vi) 
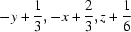
; (vii) 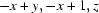.

**Fig. 1.**
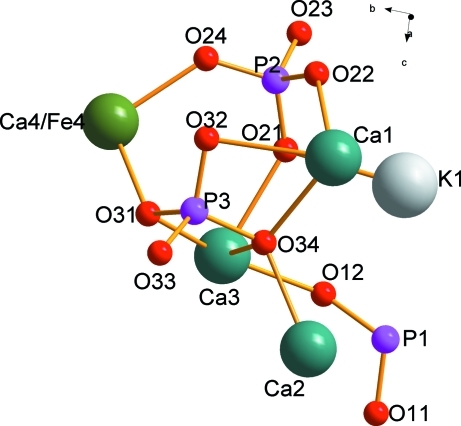
A view of the unit cell content of compound (I).

**Fig. 2.**
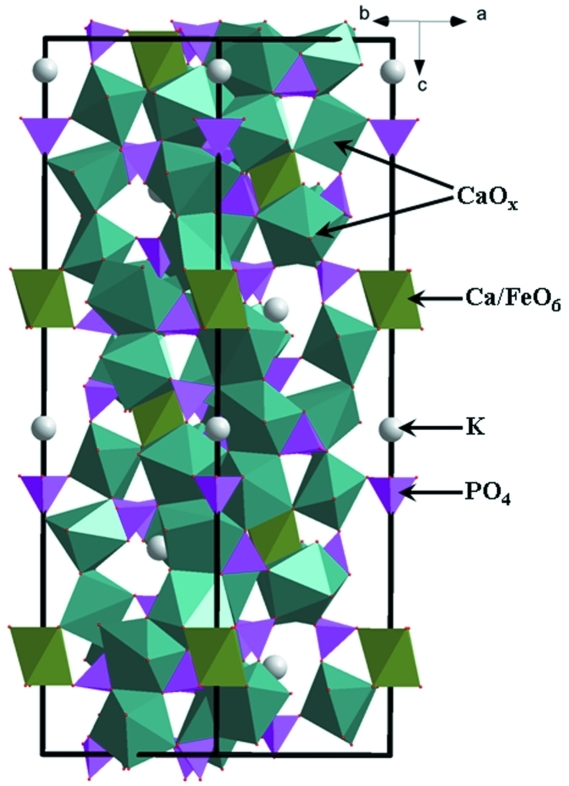
Connectivity of the metal-oxygen polyhedra and PO4 groups in (I).

**Fig. 3.**
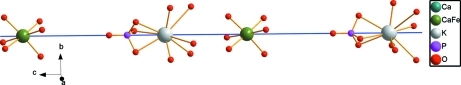
Coordination environment of the atoms in 6a position.

**Fig. 4.**
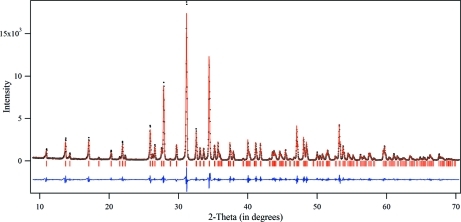
Rietveld refinement of K0.8Ca9.8Fe0.2(PO4)7. Experimental (dots), calculated (red curve) and difference (blue curve) data for 2θ range 9-72°.

## supplementary crystallographic information

### Comment

In the compound K_0.8_Ca_9.8_Fe_0.2_(PO_4_)_7_, (I), atoms Ca4/Fe4, K1, P1 and O11 are in special positions 6*a* that lie on a 3-fold rotation axis, whereas all other atoms are located in general positions 18*b* (Fig. 1).

Compound (I) might be represented as a result of an aliovalent substitution of calcium atoms in β-Ca_3_(PO_4_)_2_ (Calvo *et al.*, 1975; Yashima *et al.*, 2003) by a pair of K and Fe atoms.

[CaO*_x_*] polyhedra (two types of [CaO_8_], one of [CaO_7_] and one [(Ca/Fe)O_6_] with mixed Fe/Ca occupancy) and three different [PO_4_] tetrahedra are linked via edges and corners to built a three-dimensional framework with composition [Ca_9.8_Fe_0.2_(PO_4_)_7_]^0.8-^ (Fig. 2). The K^+^ cations are located in large closed cavities inside the framework (K1 occupancy is equal to 0.8).

For (I), Ca—O distances of [CaO_8_]- and [CaO_7_]-polyhedra (2.295 (15)-2.78 (3) Å) are close to these in previously reported isotypic compounds *s*-Ca_9_Fe(PO_4_)_7_ (2.29 (3)-2.73 (3) Å), *o*-Ca_9_Fe(PO_4_)_7_ (2.29 (3)-2.70 (4) Å) (Lazoryak *et al.*, 1996) and KCa_10_(PO_4_)_7_ (2.329 (3)- 2.76 (2) Å) (Sandström & Boström, 2006). The distances Ca/Fe—O (2.23 (4)-2.29 (3) Å) within the [(Ca/Fe)O_6_] polyhedron are close to these of the [CaO_6_] polyhedron in KCa_10_(PO_4_)_7_ (2.239 (4)-2.267 (4) Å), while they significantly differ from *d*(Fe—O) = 1.95 (3)-2.17 (3) Å in Ca_9_Fe(PO_4_)_7_.

Potassium atoms are nine-coordinated (three triples of K—O distances in the range of 2.508 (19)-3.24 (3) Å) (Fig. 3), while in KCa_10_(PO_4_)_7_ the K—O contacts vary in the range of 2.641 (3)-3.25 (4) Å .

In conclusion, compound (I) can be considered as a solid solution within the KCa_10_(PO_4_)_7_ / Ca_9_Fe(PO_4_)_7_ double system.

### Experimental

The title compound was prepared by solid state reaction from a mixture of K_2_CO_3_, CaCO_3_, Fe_2_O_3_ and NH_4_H_2_PO_4_ in the molar ratio K/Ca/Fe/P = 0.8:9.8:0.2:7.0. The reagents were finely ground in an agate mortar and then placed in a porcelain crucible. The thermal treatment was carried out in three steps. The first included preheating to 873 K to decompose the ammonium salt and carbonates. After that, the mixture was heated at 1273 K for 12 h, cooled to room temperature, reground, and held at 1373 K for 6 h. The resulting product was a pale pink powder.

### Refinement

The powder pattern was indexed in rhombohedral cell (hexagonal setting) by *Dicvol 2004* (Boultif & Louër, 2004). The structure of KCa_10_(PO_4_)_7_ (Sandström & Boström, 2006) was selected as a starting model for Rietveld refinement. Profile matching refinement was performed firstly. Then scaling factor and background were added to the refined parameters. The background was approximated using linear interpolation between a set of background points with refineable heights. A modified pseudo-Voigt function (Thompson *et al.*, 1987) was used for the profile refinement. As it was determined previously, only one position of calcium is suitable for heterovalent substitution by a three-valent 3*d*-metal. It is the octahedrally coordinated Ca4 site. Thus the iron site was placed into the Ca4 position. The occupancy of iron was fixed at 0.2 while the remaining calcium occupancy was set to 0.8. The potassium occupancy was set to 0.8 due to electroneutrality of the compound. The atomic coordinates and B_iso_ of Ca and Fe were constrained to be equal. ADPs of all P atoms were constrained to be equal as well as the ADPs of all O atoms. The value of B_iso_ for Ca4 was restrained in the range of 0.17-0.3. The value of B_iso_ for O11 was also restrained in the range of 0.2-0.3. Two distance restraints for P2—O21 and P2—O23 bonds were applied. Experimental, calculated and difference patterns after the final refinement cycle are shown in Fig. 4.

### Crystal data

**Table:**

K_0.8_Ca_9.8_Fe_0.2_(PO_4_)_7_	*D*_x_ = 3.112 Mg m^−^^3^
*M**_r_* = 1100.02	Cu *K*α radiation, λ = 1.540598 Å
Trigonal, *R*3*c*	*T* = 293 K
Hall symbol: R 3 -2"c	Particle morphology: isometric
*a* = 10.44282 (1) Å	light pink
*c* = 37.29443 (3) Å	flat_sheet, 25 × 25 mm
*V* = 3522.17 (1) Å^3^	Specimen preparation: Prepared at 293 K and 101.3 kPa
*Z* = 6	

### Data collection

**Table:**

Shimadzu LabX XRD-6000 diffractometer	Data collection mode: reflection
Radiation source: X-ray tube, X-ray	Scan method: step
graphite	2θ_min_ = 8.91°, 2θ_max_ = 99.92°, 2θ_step_ = 0.02°
Specimen mounting: glass container	

### Refinement

**Table:**

*R*_p_ = 8.711	Profile function: Thompson–Cox–Hastings pseudo-Voigt * Axial divergence asymmetry
*R*_wp_ = 11.243	131 parameters
*R*_exp_ = 4.919	4 restraints
*R*_Bragg_ = 3.849	4 constraints
*R*(*F*) = 2.48	Standard least squares refinement
χ^2^ = 5.368	(Δ/σ)_max_ = 0.001
4551 data points	Background function: Linear Interpolation between a set background points with refinable heights
Excluded region(s): undef	Preferred orientation correction: Modified March's Function

### Special details

**Table:**

Geometry. Bond distances, angles etc. have been calculated using the rounded fractional coordinates. All su's are estimated from the variances of the (full) variance-covariance matrix. The cell esds are taken into account in the estimation of distances, angles and torsion angles

### Fractional atomic coordinates and isotropic or equivalent isotropic displacement parameters (Å^2^)

**Table:**

	*x*	*y*	*z*	*U*_iso_*/*U*_eq_	Occ. (<1)
Ca1	0.3986 (5)	0.1868 (7)	0.0212 (4)	0.0022 (18)*	
Ca2	0.3922 (6)	0.1887 (10)	0.1265 (4)	0.0022 (16)*	
Ca3	0.1776 (11)	0.3817 (6)	0.0949 (5)	0.003 (2)*	
Ca4	0.33333	0.66667	0.0288 (5)	0.002 (2)*	0.80000
Fe4	0.33333	0.66667	0.0288 (5)	0.002 (2)*	0.20000
K1	0.00000	0.00000	0.0447 (5)	0.004 (4)*	0.80000
P1	0.00000	0.00000	0.1293 (5)	0.0031 (11)*	
P2	0.1351 (9)	0.3124 (6)	−0.0032 (4)	0.0031 (11)*	
P3	0.4897 (11)	0.4749 (11)	0.0609 (5)	0.0031 (11)*	
O11	0.00000	0.00000	0.1699 (8)	0.0025 (11)*	
O12	0.0071 (19)	0.1449 (14)	0.1115 (7)	0.0025 (11)*	
O21	0.0912 (15)	0.2697 (15)	0.0349 (4)	0.0025 (11)*	
O22	0.222 (2)	0.233 (2)	−0.0145 (6)	0.0025 (11)*	
O23	−0.0066 (16)	0.265 (2)	−0.0248 (5)	0.0025 (11)*	
O24	0.229 (3)	0.4728 (17)	−0.0110 (6)	0.0025 (11)*	
O31	0.408 (3)	0.567 (3)	0.0709 (7)	0.0025 (11)*	
O32	0.5039 (17)	0.4689 (16)	0.0203 (5)	0.0025 (11)*	
O33	0.6427 (19)	0.5475 (19)	0.0808 (6)	0.0025 (11)*	
O34	0.3720 (19)	0.3100 (19)	0.0752 (7)	0.0025 (11)*	

### Atomic displacement parameters (Å^2^)

**Table:**

	*U*^11^	*U*^22^	*U*^33^	*U*^12^	*U*^13^	*U*^23^
?	?	?	?	?	?	?

### Geometric parameters (Å, °)

**Table:**

Ca1—O11^i^	2.519 (10)	Ca4—O31^viii^	2.229 (35)
Ca1—O21^ii^	2.702 (13)	Fe4—O24^viii^	2.299 (30)
Ca1—O22	2.509 (26)	Fe4—O24	2.299 (30)
Ca1—O23^ii^	2.397 (20)	Fe4—O24^vii^	2.299 (30)
Ca1—O32	2.579 (17)	Fe4—O31	2.229 (35)
Ca1—O32^iii^	2.573 (23)	Fe4—O31^vii^	2.229 (35)
Ca1—O33^iii^	2.591 (27)	Fe4—O31^viii^	2.229 (35)
Ca1—O34	2.479 (28)	K1—O12	2.896 (30)
Ca2—O12^ii^	2.474 (16)	K1—O12^ii^	2.896 (30)
Ca2—O23^iv^	2.629 (26)	K1—O12^ix^	2.896 (30)
Ca2—O24^iv^	2.444 (19)	K1—O21	2.508 (19)
Ca2—O24^v^	2.484 (33)	K1—O21^ii^	2.508 (19)
Ca2—O32^v^	2.410 (23)	K1—O21^ix^	2.508 (19)
Ca2—O33^iii^	2.207 (27)	K1—O22	3.245 (26)
Ca2—O34	2.362 (29)	K1—O22^ii^	3.245 (26)
Ca3—O12	2.295 (15)	K1—O22^ix^	3.245 (26)
Ca3—O21	2.477 (22)	P1—O11	1.51 (4)
Ca3—O22^vi^	2.485 (29)	P1—O12	1.62 (2)
Ca3—O23^iv^	2.301 (25)	P1—O12^ix^	1.62 (2)
Ca3—O31	2.383 (25)	P1—O12^ii^	1.62 (2)
Ca3—O31^vii^	2.468 (36)	P2—O21	1.49 (2)
Ca3—O33^vii^	2.781 (25)	P2—O22	1.56 (2)
Ca3—O34	2.597 (27)	P2—O23	1.53 (2)
Ca4—O24^viii^	2.299 (30)	P2—O24	1.486 (17)
Ca4—O24	2.299 (30)	P3—O31	1.62 (3)
Ca4—O24^vii^	2.299 (30)	P3—O32	1.53 (3)
Ca4—O31	2.229 (35)	P3—O33	1.57 (3)
Ca4—O31^vii^	2.229 (35)	P3—O34	1.63 (2)
			
O24—Fe4—O24^vii^	82.8 (11)	O22—P2—O23	114.2 (13)
O24—Fe4—O31^vii^	101.7 (10)	O22—P2—O24	108.3 (15)
O24^viii^—Fe4—O31	101.6 (10)	O23—P2—O24	104.3 (15)
O31—Fe4—O31^viii^	75.9 (12)	O31—P3—O32	110.2 (15)
O24^vii^—Fe4—O31	175.2 (13)	O31—P3—O33	108.4 (15)
O31—Fe4—O31^vii^	75.9 (14)	O31—P3—O34	102.1 (15)
O24^viii^—Fe4—O31^viii^	99.6 (11)	O32—P3—O33	113.1 (14)
O24^viii^—Fe4—O24^vii^	82.8 (11)	O32—P3—O34	108.6 (13)
O24^viii^—Fe4—O31^vii^	175.2 (12)	O33—P3—O34	113.8 (14)
O24^vii^—Fe4—O31^viii^	101.7 (11)	O12^ix^—P1—O12^ii^	104.3 (12)
O31^viii^—Fe4—O31^vii^	75.9 (13)	O11—P1—O12^ii^	114.2 (11)
O24^vii^—Fe4—O31^vii^	99.6 (13)	O11—P1—O12	114.2 (11)
O24—Fe4—O31	99.6 (9)	O11—P1—O12^ix^	114.2 (11)
O24—Fe4—O24^viii^	82.8 (11)	O12—P1—O12^ix^	104.4 (12)
O24—Fe4—O31^viii^	175.2 (12)	O12—P1—O12^ii^	104.4 (13)
O21—P2—O22	105.8 (12)	Fe4—O24—P2	128.4 (15)
O21—P2—O23	107.5 (11)	Fe4—O31—P3	121.8 (16)
O21—P2—O24	117.0 (13)		

Symmetry codes: (i) −*x*+*y*+2/3, *y*+1/3, *z*−1/6; (ii) −*x*+*y*, −*x*, *z*; (iii) −*y*+1, *x*−*y*, *z*; (iv) *x*+1/3, *x*−*y*+2/3, *z*+1/6; (v) −*x*+*y*+1/3, *y*−1/3, *z*+1/6; (vi) −*y*+1/3, −*x*+2/3, *z*+1/6; (vii) −*x*+*y*, −*x*+1, *z*; (viii) −*y*+1, *x*−*y*+1, *z*; (ix) −*y*, *x*−*y*, *z*.
